# ASO Author Reflections: Portal Venous Glypican-3-Positive Circulating Tumor Cells Predict Microscopic Portal Vein Invasion and Survival in Hepatocellular Carcinoma

**DOI:** 10.1245/s10434-025-18459-3

**Published:** 2025-10-06

**Authors:** Daisuke Takei, Tsuyoshi Kobayashi, Hideki Ohdan

**Affiliations:** 1 Department of Gastroenterological and Transplant Surgery, Graduate School of Biomedical and Health Sciences, Hiroshima University, Hiroshima, Japan; 2 Department of Gastroenterological Surgery, Shikoku Cancer Center, National Hospital Organization, Matsuyama, Japan

## Past

Patients with hepatocellular carcinoma (HCC) frequently exhibit portal vein invasion, and microscopic portal vein invasion (mPVI) can predict early recurrence and poor survival.^1^ However, reliable preoperative identification of mPVI remains challenging, limiting ability to tailor surgical strategies and perioperative management. Circulating tumor cell (CTC) research in HCC has focused on peripheral blood samples and epithelial cellular adhesion molecule (EpCAM)-based detection, which may not fully capture the tumor biology of portal venous dissemination.^2^ Previously, the authors developed a method for detecting glypican-3 (GPC3)-positive CTCs and demonstrated its clinical utility.^3^ However, its prognostic and predictive value in portal venous blood remains unclear.

## Present

This prospective study involved 146 patients undergoing curative resection for HCC. Portal venous blood was sampled intraoperatively to quantify GPC3-positive CTCs in portal vein blood (poCTCs). The presence of poCTCs independently predicted mPVI and was significantly associated with disease-free survival and overall survival. Compared with peripheral or hepatic venous CTCs, poCTCs reflect tumor burden and biologic potential for portal dissemination more accurately. A study has shown that PD-L1-positive poCTCs are correlated with worse outcomes, suggesting immune evasion.^4^ These findings establish portal venous CTC analysis as a sensitive biomarker for risk stratification in HCC.

## Future

Minimally invasive techniques for preoperative portal venous blood sampling such as endoscopic ultrasound (EUS)-guided or percutaneous transhepatic approaches could enable broader clinical application of poCTC analysis. Monitoring PD-L1-positive CTCs may inform patient selection for immune checkpoint inhibitors and guide postoperative surveillance intensity.^5^ Integrating poCTC quantification and phenotyping into personalized treatment algorithms could refine surgical decision-making, identify adjuvant therapy candidates, and improve long-term outcomes. Multicenter and international collaborations are essential to standardize poCTC detection methods and validate their prognostic use for clinical implementation.
